# Metformin Improves Cognition by Reducing Leaky Gut and Benefiting Gut Microbiome–Goblet Cell–Mucin Axis

**DOI:** 10.1093/geroni/igaa057.438

**Published:** 2020-12-16

**Authors:** Hariom Yadav, Shokouh Ahmadi, Bo Wang, Jamie Justice, Jingzhong Ding, Dalane Kitzman, Donald McClain, Stephen Kritchevsky

**Affiliations:** 1 Wake Forest School of Medicine, Winston-Salem, North Carolina, United States; 2 North Carolina A&T State University, North Carolina, United States

## Abstract

Older adults are suffering from several aging-related illnesses including cognitive decline and effective strategies to prevent and/or treat them are lacking, because of a poor understanding of therapeutic targets. Low-grade inflammation is a key risk factor of aging-related morbidities and mortalities, and it is often higher in older adults. Although, precise reasons for increased inflammation remain unknown, however, emerging evidence indicates that abnormal (dysbiotic) gut microbiome and dysfunctional gut permeability (leaky gut) are linked with increased inflammation in older adults. However, no drugs are available to treat aging-related microbiome dysbiosis and leaky gut, and little is known about the cellular and molecular processes that can be targeted to reduce leaky gut in older adults. Here, we demonstrated that metformin, a safe FDA approved antidiabetic drug, decreased leaky gut and inflammation in older obese mice, by beneficially modulating the gut microbiota. In addition, metformin increased goblet cell mass and mucin production in the older gut, thereby decreasing leaky gut and inflammation. Mechanistically, metformin increased the goblet cell differentiation markers by suppressing Wnt signaling. Our results suggest that metformin can prevent and treat aging-related leaky gut and inflammation, by beneficially modulating gut microbiome/goblet cell/mucin biology.

